# Incidence and factors associated with burnout in radiologists: A systematic review

**DOI:** 10.1016/j.ejro.2023.100530

**Published:** 2023-10-23

**Authors:** Nader Ashraf, Muhammad Junaid Tahir, Abdullah Saeed, Mohammad J. Ghosheh, Tamara Alsheikh, Ali Ahmed, Ka Yiu Lee, Zohaib Yousaf

**Affiliations:** a College of Medicine, Alfaisal University, Riyadh 11533, Saudi Arabia; b Pakistan Kidney and Liver Institute and Research Center (PKLI & RC), Lahore 54000, Pakistan; c School of Pharmacy, Monash University, Jalan Lagoon Selatan, Bandar Sunway, Subang Jaya, Selangor, Malaysia; d Swedish Winter Sports Research Centre, Department of Health Sciences, Mid Sweden University, Östersund, Sweden; e Tower Health, Reading, PA, USA

**Keywords:** Burnout, Radiology, Stress, Workload, Prevalence

## Abstract

**Rationale and objectives:**

Burnout among physicians has a prevalence rate exceeding 50%. The radiology department is not immune to the burnout epidemic. Understanding and addressing burnout among radiologists has been a subject of recent interest. Thus, our study aims to systematically review studies reporting the prevalence of burnout in physicians in the radiology department while providing an overview of the factors associated with burnout among radiologists.

**Materials and methods:**

The search was conducted from inception until November 13th, 2022, in PubMed, Embase, Education Resources Information Center, PsycINFO, and psycArticles. Studies reporting the prevalence of burnout or any subdimensions among radiology physicians, including residents, fellows, consultants, and attendings, were included. Data on study characteristics and estimates of burnout syndrome or any of its subdimensions were collected and summarized.

**Results:**

After screening 6379 studies, 23 studies from seven countries were eligible. The number of participants ranged from 26 to 460 (median, 162; interquartile range, 91–264). In all, 18 studies (78.3%) employed a form of the Maslach Burnout Inventory. In comparison, four studies (17.4%) used the Stanford Professional Fulfillment Index, and one study (4.3%) used a single-item measure derived from the Zero Burnout Program survey. Overall burnout prevalence estimates were reported by 14 studies (60.9%) and varied from 33% to 88%. High burnout prevalence estimates were reported by only five studies (21.7%) and ranged from 5% to 62%. Emotional exhaustion and depersonalization prevalence estimates were reported by 16 studies (69.6%) and ranged from 11%−100% and 4%−97%, respectively. Furthermore, 15 studies (65.2%) reported low personal accomplishment prevalence, ranging from 14.7% to 84%. There were at least seven definitions for overall burnout and high burnout among the included studies, and there was high heterogeneity among the cutoff scores used for the burnout subdimensions.

**Conclusion:**

Burnout in radiology is increasing globally, with prevalence estimates reaching 88% and 62% for overall and high burnout, respectively. A myriad of factors has been identified as contributing to the increased prevalence. Our data demonstrated significant variability in burnout prevalence estimates among radiologists and major disparities in burnout criteria, instrument tools, and study quality.

## 1 Introduction

The World Health Organization describes burnout as a syndrome resulting from chronic unmanaged workplace stress [1]. Burnout is on the rise amongst physicians, with prevalence rates exceeding 50% [2], [3], with radiologists reporting higher levels of burnout than physicians in many other specialties [2], [4]. Maslach et al. defined burnout under three subdimensions [5]: emotional exhaustion (EE), which refers to feelings of fatigue and exhaustion of emotional resources; depersonalization (DP), which is a defense mechanism to separate oneself from work with feelings of negativism and cynicism; and reduced personal accomplishment (PA), which refers to the feelings of inadequacy or incompetency with work-related achievements [6], [7].

Burnout in healthcare can affect the functionality and working quality by increasing medical errors [8], [9], exposing the healthcare team and hospitals to malpractice lawsuits with substantial costs [10], low patient satisfaction [11], [12], and poor care delivery [13], [14]. Moreover, personal consequences like substance abuse and suicide around bound to occur due to burnout [15], [16]. What was expected to be a temporary adaption during the pandemic, work from home showed multiple advantages and will likely remain a component of the radiology departments for the long term [17]. Nonetheless, the lack of personal interactions and the many distractions associated with working remotely can increase the risk of burnout [17], [18]. Burnout is a spectrum resulting from a multitude of factors: excessive workloads, inefficient workflow, administrative obligations, work-home conflicts, lack of engagement of physicians over issues impacting their work life, organizational support systems, and leadership culture [19].

The radiology department is not immune to this epidemic. Emerging literature highlights burnout amongst radiologists, from trainees to department chairs [20], [21], [22]. It was reported that 54–72% of diagnostic and interventional radiologists exhibit burnout symptoms [23]. In the world of declining Medicare reimbursement [24], radiologists are under more pressure to maintain a high level of accuracy while dealing with a substantially higher number of cases. Moreover, longer workdays with more after-hours obligations, higher expectations for report turnaround times, competing time demands (clinical, academic, administrative), and insufficient personnel are all factors that contribute to a sensation of work overload in the radiology department [25], [26], [27]. Current practice environments may also be a contributing factor, with 75% of physicians being employed by large organizations such as academic medical centers, health maintenance organizations, large practice groups and hospitals [28]. Hence, it is more likely for radiologists to face an ineffective, obsolete, and dominant hierarchical leadership model coupled with drives toward commoditization, market consolidation, and cost containment, which may contribute to burnout [25], [28]. With the advent of PACS (Picture Archiving and Communications Systems) causing significant drop in face-to-face and telephone consultations between referring physicians and radiologists [29], there is a rise in radiologists' isolation from other health care professionals which contribute to a poor sense of PA and greater DP [27], [28]. While working remotely for radiologists has been explored as a potential mitigator of stress during the COVID-19 era, work-life balance may suffer when boundaries between work and personal life become blurred, distracting radiologists from performing their tasks [30]. In addition, while some sections are very suitable to working remotely, other sections, such as interventional radiology or pediatric radiology, have more hands-on procedures and need to remain on site [30]. Moreover, interdepartmental dynamics may cause additional stress for interventional radiologists [31]. In particular, the role of interventional radiology within big health-care systems is changing. Interventional radiologists are sometimes regarded as technicians rather than practitioners, resulting in operations being requested in the same manner as diagnostic imaging examinations. This practice immediately erodes interventionalists' autonomy to independently assess, recommend, and manage patients, as well as devalues their experience. Furthermore, seeing interventional procedures in the same light as diagnostic imaging tests has resulted in the expectation of quick service and operations, putting further strain on interventionalists.

Understanding and addressing burnout amongst radiologists is a recent subject of interest [23], [27], [31]. Therefore, we performed a systematic review to provide an overview of studies reporting the prevalence of burnout in physicians in the radiology department and the factors associated with burnout among radiologists.

## 2 Methods

This protocol was submitted with the PROSPERO database (www.crd.york.ac.uk/prospero/), the International prospective register of systematic reviews, in October 2022 with a registration ID of PROSPERO 2022 CRD42022362087. It can be accessed online at https://www.crd.york.ac.uk/prospero/display_record.php?ID=CRD42022362087. This review was conducted according to the PRISMA guidelines [32].

### 2.1 Search strategy

A local librarian searched the literature for studies exploring the prevalence of burnout in radiologists. The search strategies were created using keywords (burnout, burned out, radiologist, radiology attending, etc.) and standardized index terms. Searches were run from inception until November 13th, 2022, in MEDLINE/PubMed, Embase, Education Resources Information Center, PsycINFO, and psycArticles. We also did manual citation searching of previous reviews and meta-analyses relevant to our topic in PubMed. All citations were exported using Mendeley®, where duplicates were removed. Search strategies are provided in the supplementary material (Supplement 1).

### 2.2 Selection criteria

Studies addressing the prevalence of burnout in radiologists were included. No restrictions were made on publication time or language. Our eligibility criteria were assembled using the Patient Intervention Comparison Outcomes Study type framework [33]. The inclusion criteria consisted of the following:

Population: any sample size of male or female radiology physicians, including residents, fellows, consultants, and attendings;.

Intervention/Comparator: assessment for burnout using a well-described method with validity support from commonly accepted sources of evidence;.

Outcomes: estimates of overall burnout syndrome or any of its subdimensions;.

Study type: cross-section, observational, or prospective survey peer-reviewed studies.

We excluded studies that included non-physicians or non-radiologists (medical students, radiology technologists or nurses, radiographers, or physicians from other specialties, including radiation oncology) that did not report the prevalence of burnout for radiology physicians (separate from other personnel if it had a mixed population), that are interventional, and that are commentaries, editorials, or review papers. In addition, studies with full text not in English, qualitative data, and those with unvalidated survey instruments were excluded. If multiple versions of an article were available, only the most thorough or recent version of an article involving the same population was considered, with the former taking precedence.

### 2.3 Data extraction and management

The records were screened using the criteria mentioned above then data were extracted independently by two reviewers in duplicate (N.A.F and M.J.G) onto a standardized Excel® sheet. Any discrepancy was resolved in consultation with a third reviewer (M.J.T). Data on first author name, date of publication, country, sample demographics (mean age, gender, specialty, etc.), year(s) of the survey, the instrument used, burnout criteria classification, and prevalence estimates of overall burnout with its subdimensions were extracted from all the included studies. Additional findings deemed of interest were retrieved from the included studies, focusing on factors associated or correlated with burnout.

### 2.4 Risk-of-Bias assessment

The quality of each included nonrandomized study was evaluated using a modified version of the Newcastle-Ottawa Scale (NOS) [34], as used in a similar systematic review on burnout prevalence [3]. The modified version of NOS assessed sample representativeness and size, comparability between respondents and nonrespondents, ascertainment of burnout, and thoroughness of descriptive statistics reporting. Two reviewers evaluated each study independently (N.A.F and M.J.G). The scoring criteria is provided in the supplementary material e Appendix 2. Any differences were addressed through discussion, resolved by consensus, and, if needed, by consultation with a third reviewer (M.J.T).

### 2.5 Data analysis

Descriptive statistics using Excel® were used to analyze the extracted data. Data was reported in the form of a narrative summary and tables.

## 3 Results

### 3.1 Search results

**Fig. 1:**
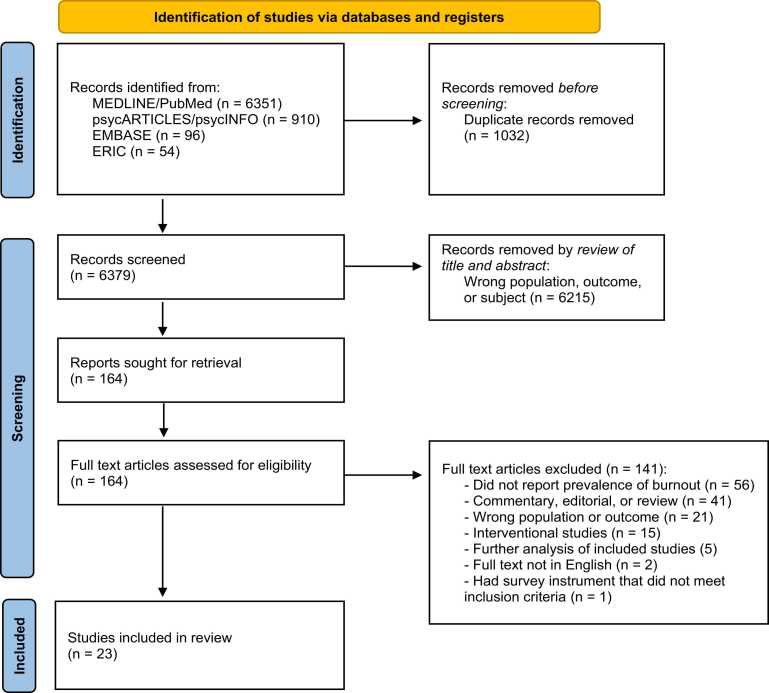
PRISMA flow diagram.

The search identified 7411 articles during screening. After 1032 duplicates were removed, 6379 articles were screened, with 6215 excluded at the title and abstract screening stage as they were deemed not eligible. This left 164 full-text articles that were assessed for eligibility, 23 of which met the criteria for final inclusion (Fig. 1).

### 3.2 Studies characteristics

Twenty-three cross-sectional studies were included, involving 4477 radiology physicians in seven countries published between 1996 and 2022 and reporting burnout prevalence estimates. The most common country of origin was the United States, with 16 studies (69.6%, n = 3428). Overall, 22 studies (95.6%) originated from high-income countries. The number of participants ranged from 26 to 460 (median, 162; interquartile range, 91–264). Nineteen studies identified the sexes of their samples, which consisted of 2437 (58.3%) males and 1740 (41.7%) females. The complete characteristics of the 22 included studies appear in Table 1.

**Table 1:** Selected Characteristics of the 23 included studies^a^.

Source	Country	Survey years	Radiology subspecialty^b^	No. of participants^c^	Male/Female, No. (%)^d^	Practice Setting	Burnout Assessment Instrument^e^	Burnout prevalence	Subscale scores or other indicators	Criteria for classification^f^
Parikh et al. (2022) [35]	United States	2021	Multiple	40 physicians	NR	Private: 100%	SPFI	Overall: 33%	PF: 43%	Burnout if an average score of greater than 1.33 from the 10 items in Questions 17 and 18
Oprisan et al. (2022) [36]	Spain	2020	Multiple	150 physicians	62/88 (41.3%/58.7%)	Tertiary care: 50% Secondary care: 22.3% Primary care: 11.5% Private:11.5% Specialist center: 4.7%	22-Item MBI-HSS	Overall: 49.3%	EE high: 52% DP high: 48% PA low: 57.3%	PA (≤33), EE (≥27) and DP (≥10) Burnout if high levels of EE plus high levels of DP and/or low levels of PA
Deshmukh et al. (2021) [37]	United States	NR	Multiple	30 physicians	19/11 (63.3%/36.7%)	Academic: 100%	1-item derived from Mini-Z	Overall: 47%	Imposter phenomenon: 83%	Burnout if reported one of the following: definitely burning out, burnout won’t go away, or completely burned out
Eisenberg et al. (2021) [38]	United States	NR	Cardiothoracic Radiology	286 physicians	110/176 (38.5%/61.5%)	Academic: 80% Private: 20%	Abbreviated 12-Item MBI	1 domain altered: 22.1% 2 domains altered: 45% 3 domains altered: 18.5%	EE high: 66.8% DP high: 79% PA low: 23%	PA (≤33), (EE (≥27) or DP (≥10) Burnout if at least one domain altered
Bundy et al. (2020) [39]	United States	2019	Interventional radiology	339 physicians	263/76 (77.6%/22.4%)	Academic: 40.1% Private: 42.8% Hybrid: 17.1%	22-Item MBI-HSS	Overall: 71.9% High burnout: 47.8%	EE high: 61.9% DP high: 54.3% PA low: 14.7%	PA (≤33), (EE (≥27) or DP (≥10) Burnout if high levels of EE or DP High burnout if both EE and DP are high
Dahmash et al. (2019) [40]	Saudi Arabia	2019	N.A.	108 residents	58/50 (53.7%/46.3%)	NR	22-Item MBI-HSS	High burnout: 24.1%	EE high: 56.5% DP high: 31.5% PA low: 64.8%	PA (≤31), EE (≥27) and DP (≥13) High burnout if all domains altered
Ferguson et al. (2020) [41]	Canada	2018	N.A.	144 residents	50/94 (34.7%/65.3%)	NR	22-Item MBI-HSS	NR	EE high: 50.7% DP high: 48.6% PA low: 36.1%	PA (≤31), (EE (≥27) or DP (≥13)
Ganeshan et al. (2020) [42]	United States	2018	Multiple	228 physicians	125/103 (54.8%/45.2%)	Academic: 100%	Abbreviated 12-Item MBI	Overall: 78.5% High burnout: 28.9%	EE high: 57.5% DP high: 72.8% PA low: 43%	PA (≤33), EE (≥27) and DP (≥10) Burnout if high levels of EE or DP High burnout if all domains altered
Ganeshan et al. (2018) [22]	United States	2018	Multiple	87 physicians	73/14 (83.9%/16.1%)	Academic: 100%	Abbreviated 12-Item MBI	Overall: 38% High burnout: 5%	EE high: 25% DP high: 24% PA low: 52%	PA (≤33), EE (≥27) and DP (≥10) Burnout if high levels of EE or DP High burnout if all domains altered
Zha et al. (2018) [43]	Canada	2018	Multiple	262 physicians	176/86 (67.2%/32.8%)	Academic: 53.4% Community: 45.4% Other: 1.2%	Abbreviated 7-Item MBI	NR	EE high: 71.8% DP high: 48.1% PA low: 17.6%	PA (≤32), EE (≥27) and DP (≥12) High burnout if all domains altered
Ayyala et al. (2018) [44]	United States	NR	Pediatric Radiology	460 physicians	226/234 (49.1%/50.9%)	Academic: 87% Private: 11% Non-hospital-based practice: 2%	Abbreviated 7-Item MBI	NR	EE high: 66% DP high: 61% PA low: 15%	PA (≤33), EE (≥27) and DP (≥10) High burnout if all domains altered
Higgins et al. (2022) [45]	United States	2017–2018	N.A.	247 residents	157/70 (69.2%/30.8%)	Academic: 100%	SPFI	Overall: 36.2	PF: 37.4% ITL: 7.6% SRI: 64.8%	PF (≥3), ITL (present if participants reported moderate, likely, or definitely), and SRI (≥16 on the PROMIS scale)
Higgins et al. (2021) [46]	United States	2017–2018	Multiple	456 physicians	285/171 (62.5%/37.5%)	Academic: 100%	SPFI	Overall: 37.4%	PF: 35.6% ITL: 33.3% SRI: 45.3%	PF (≥3), ITL (present if participants reported moderate, likely, or definitely), and SRI (≥16 on the PROMIS scale)
Giess et al. (2020) [4]	United States	2017	Multiple	162 physicians	82/80 (50.6%/49.4%)	Academic: 100%	SPFI	Overall: 35.2%	NR	Burnout if reported one of the following: definitely burning out, burnout won’t go away, or completely burned out
Chew et al. (2017) [47]	United States	2016	Musculoskeletal radiology	433 physicians	339/94 (78.3%/21.7)	Academic: 47.7% Private: 50.5% Hybrid: 1.9%	Abbreviated 7-Item MBI	Overall: 80.5% 1 domain altered: 28.2% 2 domains altered: 30.6% 3 domains altered: 21.7%	EE high: 61.7% DP high: 53.3% PA low: 39.6%	PA (≤33), EE (≥27) and DP (≥10) Burnout if at least one domain altered
Guenette et al. (2017) [48]	United States	2016	N.A.	94 residents	59/35 (62.8%/37.2%)	NR	22-Item MBI-HSS	NR	EE high: 37% DP high: 48% PA low: 50%	PA (≤32), EE (≥27) and DP (≥11) High burnout if all domains altered
Porrino et al. (2017) [49]	United States	2016	Musculoskeletal radiology	58 fellows	48/10 (82.8%/17.2%)	NR	Abbreviated 7-Item MBI	Overall: 88% 1 domain altered: 15.5% 2 domains altered: 36.2% 3 domains altered: 36.2%	EE high: 57% DP high: 67% PA low: 84%	PA (≤33), EE (≥27) and DP (≥10) Burnout if at least one domain altered
Singh et al. (2016) [50]	Australia and New Zealand	NR	Multiple	35 physicians	22/13 (62.9%/37.1%)	NR	22-Item MBI-HSS	NR	EE high: 100% DP high: 97.1% PA low: 34.3%	PA (≤31), EE (≥27) and DP (≥13) Burnout if all domains altered
Holmes et al. (2017) [51]	United States	2014	Multiple	26 physicians	NR	Tertiary academic center: 100%	22-Item MBI-HSS	Overall: 85%	NR	PA (≤33), EE (≥27) and DP (≥10) Burnout if high levels of EE or DP
McNeeley et al. (2013) [52]	United States	2012	N.A.	266 residents	194/72 (72.9%/27.1%)	NR	Abbreviated 7-Item MBI	High burnout: 62%	EE high: 53% DP high: 49%	High EE or DP (responses of at least weekly or more frequently) High burnout risk if high levels of EE or DP
Shanafelt et al. (2012) [53]	United States	2010	Multiple	216 physicians	NR	NR	22-Item MBI-HSS	Overall: 48%	NR	PA (≤33), EE (≥27) and DP (≥10) Burnout if high levels of EE or DP
Lim et al. (2009) [54]	New Zealand	NR	Multiple	136 physicians	89/47 (65.4%/34.6%)	Academic: 20% Private: 25% Hybrid: 55%	22-Item MBI-HSS	NR	Radiologists in academic practice EE high: 23% DP high: 12% PA low: 65% Radiologists in private practice EE high: 11% DP high: 4% PA low: 49%	PA (≤39), EE (≥28) and DP (≥11) Burnout if at least one domain altered
Ramirez et al. (1996) [55]	United Kingdom	1993–1994	Multiple	214 physicians	NR	NR	22-Item MBI-HSS	NR	EE high: 33% DP high: 21% PA low: 49%	Scores are considered “high” if they are in the upper third of the normative distribution, “average” if they are in the middle third, and “low” if they are in the lower third. High burnout if all domains altered

DP: depersonalization; EE: emotional exhaustion; ITL: intention to leave; MBI: Maslach Burnout Inventory; MBI-HSS: MBI–Human Services Survey; Mini Z: Zero Burnout Program Survey; NA: not applicable; NR: not reported; PA: personal accomplishment; PF: professional fulfillment; PROMIS: Patient Reported Outcomes Measurement Information System; SPFI: Stanford Professional Fulfillment Index; SRI: sleep-related impairment

a Studies are ordered according to the survey years. The publication year was referenced if survey years were not reported.

b Studies that did not specify the subspecialties involved were assumed to include participants from various specialties.

c Some participants did not answer all questions; hence, participants for one or more burnout components were lower than the total sample size

d If age and gender data for the entire population of included practicing physicians were not explicitly reported by the study; they were inferred when possible. For studies that involved mixed population (physician specialties other than radiology, nurses, radiographers, etc.), age and gender data was not included unless specified for the radiology physicians population

e If the burnout assessment method was not specified, it was inferred based on the articles or manuals the study cited.

f If the cutoff was not explicitly reported by the study, it was inferred when possible based on the articles or manuals the study cited.

### 3.3 Instruments used to assess burnout

As part of the inclusion criteria, all 23 studies had a validated measurement tool to generate these prevalence estimates. In all, 18 studies [22], [36], [38], [39], [40], [41], [42], [43], [44], [47], [48], [49], [50], [51], [52], [53], [54], [55] (78.3%) employed a form of the Maslach Burnout Inventory (MBI) [56], while 4 studies [4], [35], [45], [46] (17.4%) used the Stanford Professional Fulfillment Index (PFI) [57]. One study [37] (4.3%) used a single-item measure derived from the Zero Burnout Program survey (Physician Work Life Study or Mini Z) [58].

Ten studies (43.5%) utilized the full-length, 22-item MBI–Human Services Survey (MBI-HSS) [36], [39], [40], [41], [48], [50], [51], [53], [54], [55], intended for professionals in human services, making it appropriate for physician respondents. The MBI-HSS requires survey participants to assess how often they encounter specific feelings of burnout at work on a 7-point Likert scale, with 0 indicating “never” and 6 indicating “every day." The 22-item MBI-HSS generates scores on three subscales: nine items on EE (scores ranging from 0 to 54), five items on DP (scores ranging from 0 to 30), and eight items on PA (scores ranging from 0 to 48). A low score on PA questions while high scores on the EE and DP questions were regarded as symptoms of burnout. Furthermore, nine studies (39.1%) employed assessment tools based on full-length MBI surveys but altered in some way. Specifically, six studies (26.1%) employed single-item measures of EE and DP adapted from the MBI-HSS and validated by West et al. [59], [60] while using five-item measures of PA as described by McNeely et al. [52], making an abbreviated 7-Item MBI [43], [44], [47], [49], [52]. In contrast, three studies (13.0%) used the abbreviated 12-item MBI [22], [38], [42], first described and validated by Gabbe et al. [61], which included 5-item measures on EE, 3-item measures on DP, and 4-item measures on PA. Multiple studies adjusted the wording of some questions to improve their applicability to radiologists.

### 3.4 Prevalence of burnout and its subcomponents

The overall burnout prevalence estimates were reported by 14 studies (60.9%) and varied from 33% to 88% [4], [22], [35], [36], [37], [38], [39], [42], [45], [46], [47], [49], [51], [53]. Furthermore, the prevalence estimates of high or severe burnout were reported by only five studies (21.7%) and ranged from 5% to 62% [22], [39], [40], [42], [52]. Still, the prevalence estimates from these studies cannot be combined nor compared due to the variability in burnout assessment techniques, definitions, outcomes, and statistical heterogeneity. There were at least nine different methods of identifying physician burnout.

Even with the 18 studies (78.3%) that employed some form of MBI, there were at least seven definitions for overall and high burnout. The most common definitions were overall burnout with high levels of EE or DP, used in 5 studies [22], [39], [42], [51], [53] (21.7%), and high burnout if all three domains were altered, used in 7 studies [22], [40], [42], [43], [44], [48], [55] (30.4%). Moreover, there were at least six distinct cutoff values for the MBI subcomponents. The most common cutoff reported by ten studies (43.5%) was an EE score of at least 27, DP of at least 10, and a PA of no more than 33 [22], [36], [38], [39], [42], [44], [47], [49], [51], [53].

This heterogeneity continued with the criteria for burnout subcomponents. EE and DP prevalence estimates were reported by 16 studies (69.6%) and ranged from 11%−100% and 4%−97%, respectively. In all, 13 studies (56.5%) utilized a cutoff score of at least 27 for EE, and eight studies (34.8%) used a score of at least 10 for DP. On the other hand, 15 studies (65.2%) reported low PA prevalence, with values ranging from 14.7% to 84%. Eight studies (34.8%) used a low PA cutoff score of no more than 33.

### 3.5 Factors associated or correlated with burnout

Significant findings relevant to burnout, deemed of interest by the authors, from the included studies were collected and organized in Table 2.

**Table 2:** Findings retrieved from the included studies and deemed of interest, with particular focus on factors associated or correlated with burnout or any of its subcomponent.

Factor	Associated with burnout	Protective effect on burnout
Age	**Being older** Dahmash et al. (2019); Ganeshan et al. (2020)	
COVID-19 pandemic	Oprisan et al. (2022)	
Earlier career stage	Eisenberg et al. (2021); Bundy et al. (2020); Zha et al. (2018); Ayyala et al. (2018)	
Exercising		Dahmash et al. (2019)
Experiencing imposter phenomena	Deshmukh et al. (2021)	
Feelings of powerlessness	Porrino et al. (2017)	
Having intentions to leave	Higgins et al. (2021); Higgins et al. (2022)	
Having more on-call shifts	Dahmash et al. (2019); Ayyala et al. (2018)	
Household debt	McNeeley et al. (2013)	
Increasing residency years	Guenette et al. (2017)	Ferguson et al. (2020)
Lack of an institutional support group	Ganeshan et al. (2018)	
Lack of appreciation from patients	Ganeshan et al. (2020)	
Lack of autonomy	Ganeshan et al. (2020)	
Low chair effectiveness scores	Ganeshan et al. (2018)	
Marital status	**Being married** Dahmash et al. (2019)	
Moonlighting		McNeeley et al. (2013)
Practice level		**Attaining academic rank of professor** Ganeshan et al. (2020)
Practice size	**Lower number of faculty members** Ganeshan et al. (2018)	
Practice type (private, academic, etc.)	**Private practice** Chew et al. (2017) **Public hospital** Lim et al. (2009)	**Community radiologists** Zha et al. (2018)
Producing fewer work relative value units per year		Eisenberg et al. (2021)
Professional fulfillment		Parikh et al. (2022); Ganeshan et al. (2018); Higgins et al. (2021); Higgins et al. (2022)
Satisfaction with career choice		Dahmash et al. (2019); Ferguson et al. (2020)
Satisfaction with education-service balance in residency		Ferguson et al. (2020)
Satisfaction with evaluation methods		Dahmash et al. (2019)
Satisfaction with staff appreciation		Dahmash et al. (2019); Ganeshan et al. (2020); Ferguson et al. (2020); Higgins et al. (2022)
Satisfaction with work/life balance		Dahmash et al. (2019); Ferguson et al. (2020); Ganeshan et al. (2020); Ganeshan et al. (2018); Porrino et al. (2017); Lim et al. (2009); Higgins et al. (2022)
Sex	**Being a female** Eisenberg et al. (2021); Bundy et al. (2020); Higgins et al. (2021); Porrino et al. (2017) **Being a male** Dahmash et al. (2019)	
Sleep-related impairment	Dahmash et al. (2019); Higgins et al. (2021); Higgins et al. (2022)	
Working as radiologist of the abdomen and pelvis		Oprisan et al. (2022)
Working more hours per day/week	Eisenberg et al. (2021); Bundy et al. (2020); Dahmash et al. (2019); Ferguson et al. (2020)	

### 3.6 Risk-of-Bias assessment

Modified NOS risk-of-bias assessment of all studies showed that the majority exhibit limitations in study quality, with the majority (10 studies, 43.5%) scoring 2/5 and no study scoring 5/5 (supplementary material eTable 2). With the inclusion of several subspecialties at multiple institutions, 17 studies (73.9%) met the requirement for sample representativeness [22], [35], [36], [40], [41], [42], [43], [45], [46], [47], [48], [50], [51], [52], [53], [54], [55]. With a minimum of 300 survey participants, only five studies (21.7%) could satisfy this requirement [39], [44], [46], [47], [51]. Only one study (4.3%) demonstrated comparability between respondents and nonrespondents [53], and all studies matched the ascertainment requirements, as it was part of our inclusion criteria. Finally, 13 studies (56.5%) satisfied the descriptive statistics requirement by using appropriate and complete measures to report findings [4], [22], [35], [36], [38], [39], [40], [42], [45], [46], [47], [51], [62].

## 4 Discussion

### 4.1 Findings

Our systematic review of 23 studies involving 4477 radiology physicians in seven countries revealed significant variability in burnout prevalence estimates ranging between 33%−88% for overall burnout and 5%−62% for high or severe burnout. Various factors were thought to contribute to or correlate to burnout. Nonetheless, the considerable heterogeneity in instruments used and burnout criteria between the assessed studies made it challenging to interpret and compare the different prevalence estimates for burnout and its subcomponents. This significant variation in the research is attributable, in part, to fluctuating definitions of burnout and uncertainties about the conceptual underpinning of the burnout construct [3]. Several systematic reviews of burnout among healthcare workers reported similar findings of methodological heterogeneity [3], [63], [64], [65], [66]. Hence, there is a need for a more consistent definition of burnout with the possible application of different indicators specific to the radiology department to monitor the implementation of policy measures for radiologists’ well-being.

### 4.2 Implications

Although most studies used the full 22-item MBI, several utilized the abbreviated MBI. The abbreviated MBI was shown to have poor positive predictive value, and caution is advised on clinical correlation due to the high rates of false positives [67]. In addition, another study found that using a single-item burnout measure, as in Mini Z, did correlate sufficiently with the EE domain but not DP [68]. The cutoff scores supplied in the Maslach Burnout Inventory Manual 3rd edition are arbitrarily established on a tercile-split basis [69]. Even with Maslach backing the definition of overall burnout as high EE and either high DP or low PA [70], others have argued that low PA is incapable of predicting burnout, nor is it part of the total concept of burnout [71], [72]. The most used cutoff score for each subcomponent relates to symptoms experienced just a few times per month on average for high EE (≥27), once per month or less on average for high DP (≥10), and once per week on average for low PA (≤33) [3]. Infrequent symptoms are less likely to indicate a clinically relevant degree of burnout, resulting in prevalence estimates to indicate symptoms of burnout instead of the clinical burnout syndrome [73] . This resulted in different cutoff values, further exacerbating the inconsistencies in studies assessing burnout.

Various factors associated with burnout within the radiology department were examined within the included studies (Table 2). With the studies reporting contrasting results about certain factors, it is important to analyze such data in the context of the study’s setting and other metrics which may be influencing the findings. For instance, being a female [38], [39], [46], [49] or a male [40] was associated with burnout in certain studies, while majority of the remaining studies reported no correlation between burnout and sex.

Among the identified factors, imposter syndrome, which is increasingly common among physicians [74], [75], impairs professional progress, career success, and well-being among radiologists [37]. Correlated significantly with burnout, the imposter phenomenon is a relatively new psychological phenomena in which highly successful individuals fail to integrate their successes, resulting in chronic emotions of self-doubt and fraudulence [37]. This self-perceived incompetence can lead to increased work demands and an inability to seek help or delegate tasks, ultimately contributing to burnout. Working as a radiologist of the abdomen or pelvis was identified as a protective factor against burnout as described by Oprisan et al. [36]. One of the studies on radiology residents showed that exercising for one or more days per week was associated with a 71% lower probability of burnout [40]. With radiologists’ burnout being a public health crisis, it is crucial to combine exercise, philanthropy, and community building in a synergistic fashion to address this matter [76]. While Guenette et al. found increasing rate of burnout among more senior residents [48], Ferguson et al. found higher burnout rates among more junior residents, which can be related to the extensive depth and breadth of knowledge required in radiology [41].

McNeeley et al. observed that with increasing debt level radiologists would report higher DP and lower quality of life [52]. Financial stress and the burden of debt can create constant pressure to meet financial obligations, which can lead to increased anxiety and decreased job satisfaction. The need to work longer hours or take on additional responsibilities to manage debt can result in a higher workload and reduced personal time, ultimately contributing to burnout. Furthermore, moonlighting, which allows radiologists to diversify their experiences, maintain a sense of professional fulfillment, and potentially increase their income, has shown a protective effect against burnout among radiologists [52]. By engaging in moonlighting, radiologists can find a balance between their personal and professional lives, alleviating some of the stress associated with their primary workload and reducing the risk of burnout. Radiologists expressing intentions to leave the profession are more susceptible to burnout. The desire to leave can stem from various factors, such as an overwhelming workload, lack of control over decision-making, or dissatisfaction with the work environment. Radiologists who contemplate leaving may experience emotional exhaustion, reduced motivation, and decreased job satisfaction, which are key components of burnout. Moreover, radiologists expressing intentions to leave are more susceptible to burnout [45], [46]. The desire to leave can stem from various factors, such as an overwhelming workload, lack of control over decision-making, or dissatisfaction with the work environment. Radiologists who contemplate leaving may experience emotional exhaustion, reduced motivation, and decreased job satisfaction, which are key components of burnout.

A high risk of bias as assessed by the modified NOS was encountered. This can impact the implications and any conclusions we reach. We discovered a fluctuating response rate, with only one study demonstrating comparability between respondents and nonrespondents. The response rate is a crucial topic in survey design since it can influence outcomes on both ends. Physicians at high risk of burnout may be reluctant to respond due to their disinterest in work-related concerns and projects. Then again, physicians at risk of burnout may be more appreciative of projects dedicated to supporting emotional well-being, recognizing the necessity of addressing their work-related exhaustion and dissatisfaction.

With the multi-factorial origin of burnout in radiologists and its serious implications on quality and safety in healthcare, it is pivotal for all institutes to reduce burnout and promote health and wellness. Burnout can be addressed, and a significant recent meta-analysis showed that individual-focused and structural or organizational measures might decrease overall burnout among physicians, with a 10% drop [77]. Failure to provide well-being solutions, dedicate time to investigate solutions, or address impediments can result in expensive physician turnover and reduced capacity to cope with unfavorable or stressful events. According to the American College of Radiology Commission on Human Resources' 2018 Annual Workforce Survey, while most radiology practice leaders recognize radiologist burnout as a significant problem, only one in five leaders reported that their practices were very effective at addressing physician burnout [78]. Therefore, we recommend following the WHO guidelines on mental health at work, which provide interventions from an organizational perspective, manager and worker training perspectives, and individual perspectives for promoting positive mental health and preventing mental health conditions [79]. Furthermore, guidelines discuss interventions to be delivered to whole workforces (universal), to workers at risk of mental health conditions (selective), or to workers experiencing emotional distress or mental health conditions (indicated). It is crucial that any initiative be deep-rooted into the institutional culture and not mere department initiatives to improve wellness [80].

To address burnout in the radiology department, we recommend following Chetlen et al. overview on the various physician-directed and organization-directed interventions, highlighting the shared responsibility of healthcare organizations and individual physicians [27]. In addition, Canon et al. provide various perspectives on the implications and strategies to mitigate physician burnout in radiology [23]. As we learned from the pandemic, teleradiology is positioned favorably among radiologists, with 64.8% reporting decreased stress levels and 64% decreased workroom interruptions [81]. In addition to improvement in report turnaround time, the remote work environment enables radiologists to practice autonomy and flexibility with work-life balance, potentially mitigating burnout [82]. When it comes to stressing the significance of preserving the mental health of the healthcare staff continuously, healthcare executives and decision-makers must step up and accept long-term accountability [83], [84]. We urge radiology leaders to abide by Parikh et al. recommendations to address radiologist burnout effectively: listening to radiologists and building change, preparing the business case to radiology practices and organizations for interventions, serving as role models, and accepting their limitations on the ability to address burnout [83].

Moreover, a recent paper by Belfi et al. also addresses the burnout pandemic. It proposes a collective action to recover joy in the workplace by using P.R.A.C.T.I.C.E: Purpose, Reflection, Appreciation, Connection, Time, Inclusion, Choosing Wisely, and Embracing [85]. These elements will act as the base for the future resilient workforce.

### 4.3 Strengths and limitations

Our study is the first comprehensive systematic review of burnout prevalence among radiology physicians. Our systematic review has limitations inherent to the included studies’ methodology and design. With high methodological heterogeneity among all included studies, interpreting the results of these studies should be done with high caution. This limited our ability to holistically analyze and reach a reliable conclusion concerning the overall prevalence of burnout in radiology physicians.

### 4.4 Future directions

More research on burnout in radiology, integrating different specialties and originating from low- and middle-income countries, is needed to determine whether radiologists have a high risk of burnout while considering the differences between the healthcare systems and organizations to influence the outcomes. Based on our research, it appears appropriate to refer to the MBI-HSS (preferably the 22-item version) as the most often used approach for burnout evaluation; nonetheless, until consensus is reached, it is recommended to report multiple prevalence estimates using a range of cutoff scores. Given the limitations of MBI, we agree with Rotenstein et al. that researchers should consider using other tools, such as the Copenhagen Burnout Inventory [86], that explicitly avoid these conceptual problems and are freely available in the public domain while more strictly adhering to the Strengthening the Reporting of Observational Studies in Epidemiology guidelines ^3^. In addition, a recent comparative analysis of 770 radiology trainees showed no significant differences between MBI-HSS and Oldenburg Burnout Inventory (OLBI) in evaluating EE and DP/disengagement [87]. OLBI can prove to be a reliable and valid instrument for measuring burnout [88]. It uses only two subscales, exhaustion, and disengagement, while not acknowledging the lack of PA as part of the burnout syndrome.

## 5 Conclusion

We identified 23 studies with a high degree of heterogeneity reporting prevalence estimates on burnout among radiologists. Burnout in radiology is increasing globally, with prevalence estimates reaching 88% and 62% for overall and high/severe burnout, respectively. With a myriad of factors contributing to the increased prevalence, this data should be used as a starting point for discussion to evaluate and resolve these difficulties in the global radiology work environment. The COVID-19 pandemic created new challenges for radiologists, the psychological impact on radiologists must be acknowledged and dealt with promptly. With the modest number of studies included and the significant methodological discrepancies, further high-quality and methodologically robust studies are needed to be conducted with the standardization of burnout definition and assessment techniques.

## Funding

The authors did not receive any grant from any funding agencies.

## Ethical statement

This study is a systematic review which does not require ethical approval.

## Author statement

The idea and conceptualization were proposed by MJT and NAF. NAF, MJG, and TA formed the search strategy, and extraction was performed with the assistance of MJT and AA. Data analysis was performed by NAF. Writing of the original draft was carried out by NAF, MJG, and TA. Final review and editing were performed by ZY, AA, AS, KYL and MJT. All authors approved the final version of the draft.

## CRediT authorship contribution statement

**Tahir Muhammad Junaid:** Writing – review & editing, Data curation, Conceptualization. **Saeed Abdullah:** Writing – review & editing. **Ashraf Nader:** Writing – original draft, Formal analysis, Data curation, Conceptualization. **Ahmed Ali:** Writing – review & editing, Data curation. **Lee Ka Yiu:** Writing – review & editing. **Ghosheh Mohammad J.:** Writing – original draft, Data curation. **Alsheikh Tamara:** Writing – original draft, Data curation. **Yousaf Zohaib:** Writing – review & editing.

## Declaration of Competing Interest

The authors declare that they have no known competing financial interests or personal relationships that could have appeared to influence the work reported in this paper.
