# Supplementary material for: Incidence and factors associated with burnout in radiologists: A systematic review
Source: Eur J Radiol Open. 2023 Oct 23;11:100530. doi: 10.1016/j.ejro.2023.100530 (PMC10618688; doi:10.1016/j.ejro.2023.100530)
Supplement: Supplementary file 1 — Supplementary material [file mmc1.pdf]

Supplementary Online Content

- **eAppendix 1.** Search strategy
- **eAppendix 2.** Modified Newcastle-Ottawa Risk-of-Bias scoring guide
- **eTable 1.** Newcastle-Ottawa Risk-of-Bias scores of the 23 studies
- **eTable 2.** Summary of the Newcastle-Ottawa Risk-of-Bias scores

This supplementary material has been provided by the authors to give readers additional information about their work.

eAppendix 1. Search strategy

PubMed (MEDLINE)

Searches	Results	Type
(burnout OR “burned out” OR depersonalization or "emotional exhaustion" or burnout, professional [MESH] or emotional stress [MESH] or psychological stress [MESH] or stress, psychological [MESH] OR compassion fatigue [MESH])<br><br>AND<br><br>("attending radiologist" OR radiologist or radiologists [MESH] OR interventional radiologist OR radiology residents OR radiology fellow* OR radiology attending OR radiology consultant*)<br><br>AND<br><br>((Cohort design) OR (Cohort stud*) OR (Cohort studies [MeSH]) OR (Cross-sectional analysis) OR (Cross-sectional design) OR (Cross-sectional stud*) OR (Cross-sectional studies [MeSH]) OR (Epidemiologic stud*) OR (Epidemiologic studies [MeSH]) OR (Incidence) OR (Longitudinal design) OR (Longitudinal stud*) OR (Meta-analy*) OR (Meta-analysis [Publication Type]) OR (Observational stud*) OR (Population stud*) OR (Prevalence) OR (Prospective design) OR (Prospective stud*) OR (Prospective studies [MeSH]) OR (Retrospective design) OR (Retrospective stud*) OR (Retrospective studies [MeSH]) OR (Review) OR (Review [Publication Type]))	**6,351**	Advanced

Embase

Searches	Results	Type
(burnout OR “burned out” OR depersonalization or "emotional exhaustion" or burnout, professional [MESH] or emotional stress [MESH] or psychological stress [MESH] or stress, psychological [MESH] OR compassion fatigue [MESH])<br><br>AND<br><br>("attending radiologist" OR radiologist or radiologists [MESH] OR interventional radiologist OR radiology residents OR radiology fellow* OR radiology attending OR radiology consultant*)<br><br>AND<br><br>((Cohort design) OR (Cohort stud*) OR (Cohort studies [MeSH]) OR (Cross-sectional analysis) OR (Cross-sectional design) OR (Cross-sectional stud*) OR (Cross-sectional studies [MeSH]) OR (Epidemiologic stud*) OR (Epidemiologic studies [MeSH]) OR (Incidence) OR (Longitudinal design) OR (Longitudinal stud*) OR (Meta-analy*) OR (Meta-analysis [Publication Type]) OR (Observational stud*) OR (Population stud*) OR (Prevalence) OR (Prospective design) OR (Prospective stud*) OR (Prospective studies [MeSH]) OR (Retrospective design) OR (Retrospective stud*) OR (Retrospective studies [MeSH]) OR (Review) OR (Review [Publication Type]))	**96**	Advanced

ERIC, PsycINFO, and PsycARTICLES

Searches	Results	Type
(burnout OR “burned out” OR depersonalization or "emotional exhaustion" or burnout, professional [MESH] or emotional stress [MESH] or psychological stress [MESH] or stress, psychological [MESH] OR compassion fatigue [MESH])<br><br>AND<br><br>("attending radiologist" OR radiologist or radiologists [MESH] OR interventional radiologist OR radiology residents OR radiology fellow* OR radiology attending OR radiology consultant*)<br><br>AND<br><br>((Cohort design) OR (Cohort stud*) OR (Cohort studies [MeSH]) OR (Cross-sectional analysis) OR (Cross-sectional design) OR (Cross-sectional stud*) OR (Cross-sectional studies [MeSH]) OR (Epidemiologic stud*) OR (Epidemiologic studies [MeSH]) OR (Incidence) OR (Longitudinal design) OR (Longitudinal stud*) OR (Meta-analy*) OR (Meta-analysis [Publication Type]) OR (Observational stud*) OR (Population stud*) OR (Prevalence) OR (Prospective design) OR (Prospective stud*) OR (Prospective studies [MeSH]) OR (Retrospective design) OR (Retrospective stud*) OR (Retrospective studies [MeSH]) OR (Review) OR (Review [Publication Type]))	**964**	Advanced

eAppendix 2. Modified Newcastle-Ottawa risk-of-bias scoring guide

(1) Representativeness of the sample:

1 point: Population contained multiple specialties at multiple institutions.

0 points: Population contained either a single specialty, a single institution, or both.

(2) Sample size:

1 point: Sample size was ≥300 participants.

0 points: Sample size was <300 participants.

(3) Non-respondents:

1 point: Comparability between respondent and non-respondent characteristics was established, or the response "rate" was 95% or greater.

0 points: The comparability between respondents and non-respondents was unsatisfactory, the response "rate" was unsatisfactory, or there was no description of the response "rate" or the characteristics of the responders or non-responders.

(4) Ascertainment of burnout:

1 point: Well described and/or validated measurement tool, *e.g.*, the MBI.

0 points: Poorly described measurement tool of uncertain validity or non-validated single-question screening tool.

(5) Quality of descriptive statistics reporting:

1 point: Reported descriptive statistics to describe the population (*e.g.*, age, sex) with proper measures of dispersion (*e.g.*, mean, standard deviation).

0 points: Descriptive statistics were not reported, were incomplete, or did not include proper measures of dispersion.

Note: This scale assesses quality in several domains: sample representativeness and size, comparability between respondents and non-respondents, ascertainment of burnout, and statistical reporting quality.

eTable 1. Newcastle-Ottawa Risk-of-Bias scores of the 23 studies

Source	Representativeness	Sample Size	Non-respondents	Ascertainment	Descriptive Statistics
Parikh et al (2022)[1]	1	0	0	1	1
Oprisan et al (2021)[2]	1	0	0	1	1
Deshmukh et al (2021)[3]	0	0	0	1	0
Eisenberg et al (2021)[4]	0	0	0	1	1
Bundy et al (2020)[5]	0	1	0	1	1
Dahmash et al (2019)[6]	1	0	0	1	1
Ferguson et al (2020)[7]	1	0	0	1	0
Ganeshan et al (2020)[8]	1	0	0	1	1
Ganeshan et al (2018)[9]	1	0	0	1	1
Zha et al (2018)[10]	1	0	0	1	0
Ayyala et al (2018)[11]	0	1	0	1	0
Higgins et al (2022)[12]	1	0	0	1	1
Higgins et al (2021)[13]	1	1	0	1	1
Giess et al (2020)[14]	0	0	0	1	1
Chew et al (2017)[15]	1	1	0	1	1
Guenette et al (2017)[16]	1	0	0	1	0
Porrino et al (2017)[17]	0	0	0	1	0
Singh et al (2016)[18]	1	0	0	1	0
Holmes et al (2017)[19]	1	1	0	1	1
McNeeley et al (2013)[20]	1	0	0	1	0
Shanafelt et al (2012)[21]	1	0	1	1	1
Lim et al (2009)[22]	1	0	0	1	0
Ramirez et al (1996)[23]	1	0	0	1	0

eTable 2. Summary of the Newcastle-Ottawa Risk-of-Bias scores (n = 23)

Domain	No. of studies (%)
**Representativeness**	
0 points	6 (26.1%)
1 point	17 (73.9%)
**Sample Size**	
0 points	18 (78.3%)
1 point	5 (21.7%)
**Non-respondents**	
0 points	22 (95.7%)
1 point	1 (4.3%)
**Ascertainment**	
0 points	0 (0%)
1 point	23 (100%)
**Descriptive Statistics**	
0 points	10 (43.5%)
1 point	13 (56.5%)
**Total Newcastle-Ottawa Score**	
0 points	0 (0%)
1 point	2 (8.7%)
2 points	10 (43.5%)
3 points	7 (30.4%)
4 points	4 (17.4%)
5 points	0 (0%)

eReferences

1. Parikh JR, van Moore A, Mead L, Bassett R, Rubin E. Prevalence of burnout in private practice radiology leaders. *Clin Imaging*. 2022;92:1-6. doi:10.1016/j.clinimag.2022.08.014

2. Oprisan A, Baettig-Arriagada E, Baeza-Delgado C, Martí-Bonmatí L. Prevalence of burnout syndrome during the COVID-19 pandemic and associated factors. *Radiología (English Edition)*. 2022;64(2):119-127. doi:10.1016/j.rxeng.2021.09.009

3. Deshmukh S, Shmelev K, Vassiliades L, Kurumety S, Agarwal G, Horowitz JM. Imposter phenomenon in radiology: incidence, intervention, and impact on wellness. *Clin Imaging*. 2022;82:94-99. doi:10.1016/j.clinimag.2021.11.009

4. Eisenberg RL, Sotman TE, Czum JM, Montner SM, Meyer CA. Prevalence of Burnout Among Cardiothoracic Radiologists. *J Thorac Imaging*. 2021;36(1):57-64. doi:10.1097/RTI.0000000000000540

5. Bundy JJ, Hage AN, Srinivasa RN, et al. Burnout among Interventional Radiologists. *Journal of Vascular and Interventional Radiology*. 2020;31(4):607-613.e1. doi:10.1016/j.jvir.2019.06.002

6. bin Dahmash A, Alorfi FK, Alharbi A, Aldayel A, Kamel AM, Almoaiqel M. Burnout Phenomenon and Its Predictors in Radiology Residents. *Acad Radiol*. 2020;27(7):1033-1039. doi:10.1016/j.acra.2019.09.024

7. Ferguson C, Low G, Shiau G. Burnout in Canadian Radiology Residency: A National Assessment of Prevalence and Underlying Contributory Factors. *Canadian Association of Radiologists Journal*. 2020;71(1):40-47. doi:10.1177/0846537119885672

8. Ganeshan D, Rosenkrantz AB, Bassett RL, Williams L, Lenchik L, Yang W. Burnout in Academic Radiologists in the United States. *Acad Radiol*. 2020;27(9):1274-1281. doi:10.1016/j.acra.2019.12.029

9. Ganeshan D, Wei W, Yang W. Burnout in Chairs of Academic Radiology Departments in the United States. *Acad Radiol*. 2019;26(10):1378-1384. doi:10.1016/j.acra.2018.12.006

10. Zha N, Patlas MN, Neuheimer N, Duszak R. Prevalence of Burnout among Canadian Radiologists and Radiology Trainees. *Canadian Association of Radiologists Journal*. 2018;69(4):367-372. doi:10.1016/j.carj.2018.05.005

11. Ayyala RS, Ahmed FS, Ruzal-Shapiro C, Taylor GA. Prevalence of Burnout Among Pediatric Radiologists. *Journal of the American College of Radiology*. 2019;16(4):518-522. doi:10.1016/j.jacr.2018.08.016

12. Higgins MCSS, Siddiqui AA, Kosowsky T, et al. Burnout, Professional Fulfillment, Intention to Leave, and Sleep-Related Impairment among Radiology Trainees across the United States (US): A Multisite Epidemiologic Study. *Acad Radiol*. 2022;29:S118-S125. doi:10.1016/j.acra.2022.01.022

13. Higgins MCSS, Nguyen MT, Kosowsky T, et al. Burnout, Professional Fulfillment, Intention to Leave, and Sleep-Related Impairment Among Faculty Radiologists in the United States: An Epidemiologic Study. *Journal of the American College of Radiology*. 2021;18(9):1359-1364. doi:10.1016/j.jacr.2021.04.005

14. Giess CS, Ip IK, Gupte A, et al. Self-reported Burnout: Comparison of Radiologists to Nonradiologist Peers at a Large Academic Medical Center. *Acad Radiol*. 2022;29(2):277-283. doi:10.1016/j.acra.2020.10.013

15. Chew FS, Mulcahy MJ, Porrino JA, Mulcahy H, Relyea-Chew A. Prevalence of burnout among musculoskeletal radiologists. *Skeletal Radiol*. 2017;46(4):497-506. doi:10.1007/s00256-017-2578-9

16. Guenette JP, Smith SE. Burnout: Prevalence and Associated Factors Among Radiology Residents in New England With Comparison Against United States Resident Physicians in Other Specialties. *American Journal of Roentgenology*. 2017;209(1):136-141. doi:10.2214/AJR.16.17541

17. Porrino J, Mulcahy MJ, Mulcahy H, Relyea-Chew A, Chew FS. Emotional Wellness of Current Musculoskeletal Radiology Fellows. *Acad Radiol*. 2017;24(6):682-693. doi:10.1016/j.acra.2016.12.024

18. Singh N, Knight K, Wright C, et al. Occupational burnout among radiographers, sonographers and radiologists in Australia and New Zealand: Findings from a national survey. *J Med Imaging Radiat Oncol*. 2017;61(3):304-310. doi:10.1111/1754-9485.12547

19. Holmes EG, Connolly A, Putnam KT, et al. Taking Care of Our Own: A Multispecialty Study of Resident and Program Director Perspectives on Contributors to Burnout and Potential Interventions. *Academic Psychiatry*. 2017;41(2):159-166. doi:10.1007/s40596-016-0590-3

20. McNeeley MF, Perez FA, Chew FS. The Emotional Wellness of Radiology Trainees. *Acad Radiol*. 2013;20(5):647-655. doi:10.1016/j.acra.2012.12.018

21. Shanafelt TD, Boone S, Tan L, et al. Burnout and Satisfaction With Work-Life Balance Among US Physicians Relative to the General US Population. *Arch Intern Med*. 2012;172(18):1377. doi:10.1001/archinternmed.2012.3199

22. Lim R, Pinto C. Work stress, satisfaction and burnout in New Zealand radiologists: Comparison of public hospital and private practice in New Zealand. *J Med Imaging Radiat Oncol*. 2009;53(2):194-199. doi:10.1111/j.1754-9485.2009.02063.x

23. Ramirez AJ, Graham J, Richards MA, Gregory WM, Cull A. Mental health of hospital consultants: the effects of stress and satisfaction at work. *The Lancet*. 1996;347(9003):724-728. doi:10.1016/S0140-6736(96)90077-X
